# Metabolic bone imaging and its relationship with biomechanics

**DOI:** 10.1016/j.ostima.2024.100242

**Published:** 2024-07-13

**Authors:** Ananya Goyal, Lauren Watkins, Olivia Bruce, Anthony Gatti, Feliks Kogan

**Affiliations:** a Stanford University, Department of Radiology, Stanford, CA United States; b Stanford University, Department of Bioengineering, Stanford, CA United States

**Keywords:** PET-MRI, Bone metabolism, Mechanical loading, Joint function

## Abstract

**Objective:**

This mini review delves into the mechanisms of [^18^F]Sodium Fluoride positron emission tomography ([^18^F]NaF PET), which, by interrogating areas of newly mineralizing bone, provides a valuable tool to study the joint response to loading and areas of altered whole-joint function in osteoarthritis (OA).

**Design:**

The review consolidates and discusses findings from both preclinical and clinical studies that utilize [^18^F]NaF PET to evaluate the bone response to various loading paradigms. It also briefly reviews technical considerations for PET imaging and discusses its strong potential as a tool in the quest to understand bone metabolism in the context of loading and osteoarthritis.

**Results:**

While considering previous studies, technical considerations and potential new applications of this methodology are also discussed. [^18^F]NaF PET/MRI reveals localized, load-related bone responses after exercise, providing insights into early OA progression. In human studies, significant increases in tracer uptake are observed in areas affected by OA pathology, driven by bone perfusion and blood volume. Future work to examine the relationship between metabolic bone response to exercise and the bone loading environment is needed.

**Conclusions:**

Integrating [^18^F]NaF PET/MRI with advanced biomechanical modeling holds promise for guiding clinical management of OA, primarily by examining the relationship between bone, soft tissues of the joint, and loading forces.

## Introduction

Joint mechanics and loading are important factors in the onset and progression of osteoarthritis (OA). Exercise and normal joint loading are thought to be beneficial for OA and joint health. On the other hand, excessive mechanical forces can be pathologic and lead to joint degeneration [1,2]. Defining “excessive” can be difficult as it is likely to change with age and the degenerative status of the joint. Joint underloading is similarly associated with degenerative changes in the knee, such as cartilage thinning [3]. Musculoskeletal models can simulate human motion and evaluate muscle and joint forces. However, evaluation of the tissue level response to these forces is challenging.

Imaging is a valuable tool for studying the response of joint tissues to loading. Magnetic resonance imaging (MRI) has been used to examine compositional changes in soft tissues like cartilage and the meniscus [4] in response to loading but is limited in its ability to detect the loading response of bone. Positron emission tomography (PET) imaging of metabolic bone changes in the joint in response to exercise is a new approach to understanding the acute effects of mechanical loading in the joint. [^18^F]Sodium Fluoride ([^18^F]NaF) is a PET radiotracer that interrogates areas of newly mineralizing bone and correlates the uptake with bone histomorphometry, allowing the study of metabolic bone activity in vivo [5,6]. Unlike most joint tissues, bone is a dynamic, well-vascularized tissue that can quickly respond to external mechanical stimuli. In the context of OA, changes in the function of subchondral bone may precede changes in cartilage [7,8]. The ability to detect these changes may assist in early diagnosis and intervention. The metabolic response of bone to loading may thus reveal focal increases in bone loading, which may indicate areas of altered whole-joint function in OA. New insights have shown that acute loading alters the bone physiology, thereby affecting [^18^F]NaF uptake [3]. In this mini review, we outline the mechanisms of [^18^F]NaF uptake and some recent work on [^18^F]NaF uptake before and after exercise. We also discuss technical considerations and look forward to new applications of this methodology.

### Mechanisms of [^18^F] sodium fluoride uptake

**Fig. 1:**
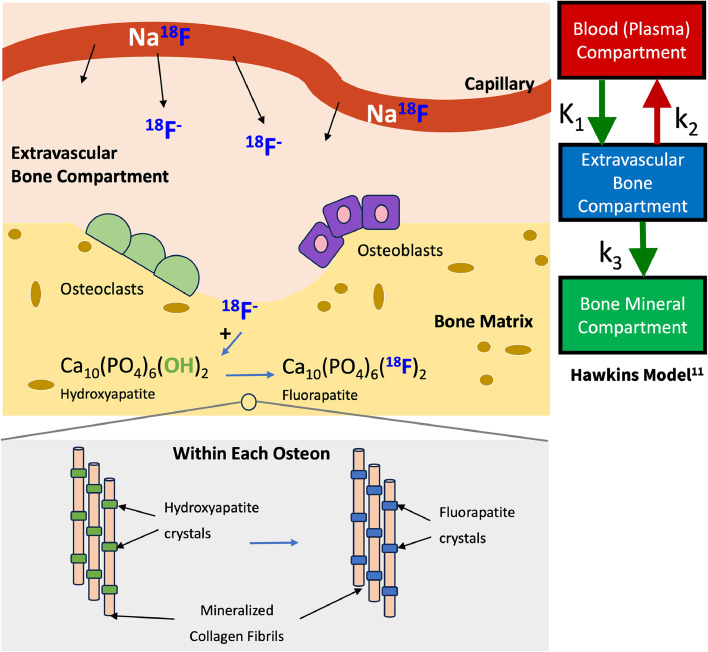
Mechanism of [^18^F]NaF PET uptake from plasma into the bone matrix, portrayed alongside the Hawkins tracer kinetic model [11].

**Fig. 2:**
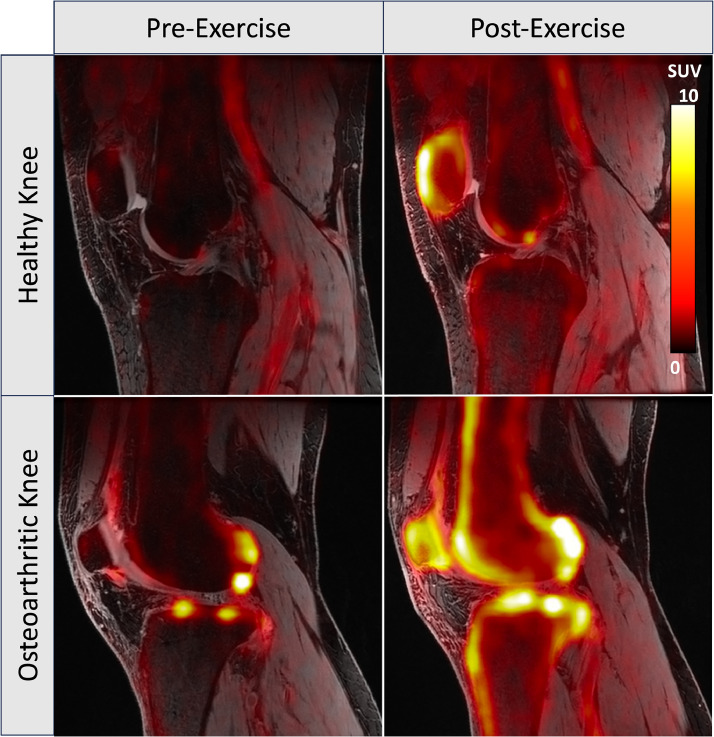
Representative fused [^18^F]NaF PET-MR images of a healthy knee (top row, 29-year-old male) and a knee with osteoarthritis (bottom row, 42-year-old male). The baseline (“Pre-Exercise”) images are shown on the left, while images acquired after a stair-climbing activity (“Post-Exercise”) are shown on the right. Both knees show increased uptake after the stair-climbing exercise, particularly in the anterior patella and the tibial tuberosity, which undergo large stresses from the patellar tendon. In the OA knee, however, multiple focal areas of increased uptake are observed, both at baseline and after exercise, representative of underlying structural pathology associated with OA.

[^18^F]NaF is a positron-emitting radiotracer used for skeletal imaging, primarily as a marker of bone turnover. Uptake of [^18^F]NaF in bone occurs due to ionic chemisorption, where fluoride (^18^F^−^) ions replace hydroxyl (OH^−^) ions, thereby converting hydroxyapatite to fluorapatite [5]. To reach the bone interior, ^18^F^−^ ions pass from the plasma to the extravascular fluid space driven by blood flow, which is usually the rate-limiting factor in [^18^F]NaF uptake. From there, the ^18^F^−^ ions pass onto the shell of bound water surrounding individual hydroxyapatite crystals. These processes occur very rapidly (within seconds to minutes), after which the tracer has become part of the bone, and clearance of the tracer from the bone is minimal. From there, bone formation continues as the ions move onto the crystal surface (within hours) and finally, into the crystal interior (within days to weeks) [10] (Fig. 1). High [^18^F]NaF uptake is thus driven by any processes that increase the exposed bone crystal surface and/or bone blood flow. Importantly, deposition of new bone mineral is not required for ^18^F^−^ uptake. Areas of osteoclastic activity are visualized just as well as areas of osteoblastic activity. However, conditions impacting bone blood perfusion, such as aging-related changes in marrow composition and osteoporosis, may lead to differences and inaccuracies in measured tracer uptake. (Fig. 2)

[^18^F]NaF tracer uptake and activity in a specific bone region may be measured in multiple ways. Standardized uptake values (SUV) provide a static measure of the tracer activity concentration [kBq/mL] normalized to the decay-corrected amount of injected tracer [kBq] and the weight of the patient. SUV is a simple method to express uptake and has been shown to be reproducible and reliable although dependent on the availability of the tracer at any particular site. As tracer distribution will vary between subjects, SUV provides only a relative measure of tracer uptake. Alternatively, dynamic PET scanning, which takes into account tracer delivery and uptake rates, may be used to extract quantitative metrics related to tracer delivery (bone perfusion – K_1_), extraction of delivered tracer to the bone versus clearance back to plasma (extraction fraction), as well as a quantitative measure of ^18^F^−^ movement from the plasma into the bone (K_i_) [11](Table 1). These kinetic parameters show precision similar to SUV in terms of repeatability, with the parameter K_i_ having a higher sensitivity to treatment response [12].

**Table 1:** Overview of PET tracer uptake and kinetic measures.

PET Parameter	**Definition**	**Calculation**
Standardized Uptake Value (SUV)	Static measure of the tracer activity concentration normalized to the decay-corrected amount of injected tracer and the weight of the patient.	$SUV\;\left[\frac{g}{mL}\right]=\frac{Tracer\;activity[\frac{kBq}{mL}]\;}{Injected\;dose\;[kBq]}*Patient\;weight\;\left[g\right]$
Bone Perfusion (K_1_)	Rate of transit of the ^18^F^−^ ions from plasma to the extravascular bone compartment	Kinetic parameter estimated from Hawkins tracer kinetic model[11] [mL min-1 mL-1]
Extraction Fraction	Fraction of extravascular ^18^F^−^ ions binding to the bone matrix	$Ex\;Frac=\;\frac{k_{3}}{k_{2}+k_{3}}$ $k_{2}$: tissue clearance [min^−1^] & $k_{3}$: mineralization [min^−1^] from Hawkins model[11]
Plasma Clearance or Total Bone Mineralization (K_i_)	Total rate of fluoride clearance from plasma to the bone matrix	${K_{i}}^{NLR}\;\left[\frac{mL}{min\;mL}\right]=K_{1}*Ex\;Frac$ (Hawkins Model[11] – Nonlinear Regression Fitting) ${K_{i}}^{PAT}$: K_i_ of [^18^F]NaF can also be determined using Patlak graphical analysis

### Preclinical models of bone response to loading

Preclinical models have been used to elucidate the mechanisms of [^18^F]NaF uptake in bone and the timing of bone metabolic responses following acute loading [[13], [14], [15]]. Studies used an established in vivo rat forelimb compression-loading model where the ulna is loaded cyclically in force control to predetermined displacement or stiffness loss thresholds, which correspond with microdamage and bone formation at the mid-diaphysis [16,17]. Using this approach, Li et al. observed elevated [^18^F]NaF uptake at the mid-diaphysis of the ulna by approximately 53 % and 60 % at one and twelve days following loading, respectively, relative to baseline scans [13]. The PET image hotspots spatially corresponded to enhanced radiotracer accumulation measured with autoradiography and to histologically observed regions of microdamage; these findings indicate that elevated [^18^F]NaF localizes to bone microdamage and subsequent remodeling. Similarly, Silva et al. observed elevated SUV beginning within 4 h of a single session of cyclic compressive loading to sub fracture (50–120 % increase relative to the unloaded limb), peaking after four to nine days (75–185 % increase relative to the unloaded limb), and returning to baseline after three weeks [15]. This increased uptake was proportional to the level of mechanical fatigue. Furthermore, histology and kinetic modeling of [^18^F]NaF indicate that the increased uptake observed in the first week following acute loading may be attributed to a combination of increased bone surface area due to microcracks (within four hours, onward), increased perfusion (at one day, onward), and woven bone formation (at 3 days, onward) [14,15,17].

### Human studies

Recent work has explored the acute bone metabolic response to loading in human cohorts, where exercise is performed between two consecutive radiotracer injections and dynamic PET scans. The second radiotracer injection and dynamic scan begin immediately after the exercise is completed. Residual tracer activity from the first scan is accounted for in determining tracer uptake in the second scan. SUV and kinetic parameters of tracer distribution and uptake into the bone are then calculated for both baseline and post-exercise scans.

Using this protocol with a population of asymptomatic volunteers, Haddock et al. observed that 100 repetitions of a step-up (right leg) and drop-down (left leg) exercise induced significant increases in SUV and K_i_, measures of total tracer uptake in the bone. This was driven primarily by increases in bone perfusion (K_1_) and blood volume [9]. Additionally, differences were observed between legs that performed step-up and drop-down exercises and within varying regions of the knee. Similar results were observed by Watkins et al. in OA patients who performed a single-leg knee extension exercise with 50 % body weight [18]. In OA patients, large changes in uptake were observed in areas of bone with osteophytes, bone marrow lesions, and adjacent cartilage loss.

In addition to disease-related changes in the metabolic bone response to load, results from both studies suggest that [^18^F]NaF may reflect a localized and load-dependent metabolic bone response. The femur, tibia, and patella all experienced increases in SUV, K_i_, and K_1_ and decreases in extraction fraction after exercise; however, the largest changes in tracer uptake were observed in the patella, which experiences high quadriceps forces during step-up activity and during squatting. Additionally, pre and post exercise changes were significantly smaller for patellae in the drop-down leg, where there is less quadriceps activation. Kinetic modeling of dynamic [^18^F]NaF showed that increased total bone uptake of ^18^F^-^ ions (K_i_) was driven by a large increase in bone perfusion (K_1_) while the extraction fraction decreased, suggestive of a hyperemia effect related to exercise similar to that seen in [^18^F]FDG PET studies of muscle. Relative increases in uptake and bone perfusion rates were observed for all bone types but were greater in regions of subchondral (2–9 cm^2^) and trabecular (16–32 cm^3^) bone than in cortical bone (9–18 cm^3^) [9,19]. In both asymptomatic and OA knees, however, there were numerous focal areas of elevated response to exercise that were not observed in the surrounding bone (change in SUV > 3). While the majority of these regions represented areas of high uptake before exercise and many were co-localized to structural pathology in OA patients, some focal areas were unremarkable on PET and MRI at rest and appeared only after exercise. This shows that the mechanisms leading to an altered metabolic bone response to loading are locally regulated and may not necessarily reflect a systemic increase in blood flow from exercise. Two years after this initial exercise study, five OA participants returned for an MRI-only exam; 17 of the 22 knee regions with focal increases in bone uptake after exercise at the initial visit showed progression in the MRI Osteoarthritis Knee Score (MOAKS) [18]. This suggests that [^18^F]NaF uptake in relation to acute exercise may provide insights into early OA pathogenesis and disease progression.

Further work is necessary to examine the relationship between the metabolic bone response to exercise and the bone loading environment. Areas of high metabolic bone response to acute loading may reflect individual differences in geometry, kinetics/kinematics, or loading patterns, thus changing gross loading magnitude. Alternatively, the response may be due to altered mechanical function in cartilage or meniscus caused by tissue structural, microstructural, or compositional changes resulting in focally high loading in the underlying bone. Thus, [^18^F]NaF may serve as an independent marker of whole-joint function, and it may also increase understanding of the role of cartilage or meniscal microstructure or composition, as measured with MRI, on whole-joint function.

### Technical considerations

As described earlier, current protocols for imaging of the metabolic bone response to acute loading involve two sequential PET scans. Careful consideration is recommended to reduce exposure to ionizing radiation; however, the dose per scan described in this review (∼2.5mCi/injection or ∼5mCi/scan (2 injections)) [9,18] is lower than clinical guidelines for doses used in PET bone imaging (∼7mCi) [20]. Multimodal PET-MRI systems provide an optimal imaging system for this approach as MRI provides excellent contrast for studying joint structure and microstructure and is also non-ionizing. Morever, these methods are transferable to PET/CT systems, with the effective dose from CT being relatively small for joints of the extremities such as the knee. Compared to PET/MRI, PET/CT may prove more beneficial for evaluating bone density and fine anatomical details, and for imaging near metal implants. New whole-body or large field of view PET/CT systems may enable the evaluation of multiple joints as might new approaches to kinetic modeling in multiple PET-bed positions.

Kinetic parameters of tracer uptake are quantitative and reproducible measures sensitive to both tracer delivery and the total metabolic bone uptake, but they require long dynamic acquisitions and extensive post-processing, including separating uptake from multiple injections of the same radiotracer for evaluating the joint response to loading [18]. Techniques to quantify tracer uptake from shorter scans have been developed [19,21] and may prove useful in the future. Simplified protocols using static PET scans and SUV metrics or dynamic information at later timepoints have also shown promise and may be sufficient to examine acute bone loading responses in some specific contexts [21].

These approaches also lead to considerations regarding the scanning of “resting” bone metabolism in the context of osteoarthritis. Studies have shown that subchondral bone metabolism is elevated in OA [22] and is related to adjacent cartilage structure and microstructure [23] as well as synovitis [24]. The sensitivity of bone [^18^F]NaF uptake to acute loading suggests that care must be taken to limit or standardize pre-scan loading in these studies, such as avoiding intensive physical activities 48 h before scans. While it may be that loading simply accentuates areas of high metabolic bone activity, it is important to ensure that subjects’ activity before the scans does not influence results. In the meantime, it is hoped that ongoing work will differentiate the mechanisms of resting metabolic bone activity from the response to various exercise loading paradigms.

### Future work

[^18^F]NaF PET shows strong potential as a tool in the quest to understand bone metabolism in the context of loading. Recent studies have begun to examine the relative contributions of loading forces, bone metabolism, and structural properties of bone and cartilage by linking finite element analysis and PET imaging of bone. In studies of the hip and jaw, maximum SUV was correlated with areas of stress concentration predicted from finite element analysis [25,26]. In the context of acute loading response, initial work by Gatti et al. found that tibial strains after repeated drop-landing and changes in [^18^F]NaF uptake were positively correlated and greatest in the distal medial tibial diaphysis [27]. Musculoskeletal models that incorporate muscle-driven simulations of exercise movements could also elucidate relationships between bone metabolic activity and muscle forces or contact pressures applied to bone. Techniques linking biomechanical models with PET imaging are promising directions for future work to better understand the relationships between bone metabolism and the loading environment. Additionally, the effects of interventional strategies such as gait retraining, orthotic devices, and weight management, that alter joint loading may also provide clinical guidance on osteoarthritis mitigation methods.

Beyond [^18^F]NaF, numerous other PET radiotracers are available for OA imaging. [^18^F]FDG can image glucose metabolism in inflammatory and infectious aspects of OA, and its uptake correlates with the severity of OA symptoms. [^18^F]FEPPA and [^11^C](*R*)-PK11195 are more specific markers for inflammation than [^18^F]FDG and target activated macrophages at sites of inflammation. Lastly, [^18^F]FAPPI is a marker of activated fibroblasts and can be used to image tissue remodeling sites associated with arthritis and fibrosis [28].

## Conclusion

Understanding how joint mechanics and loading affect OA onset and progression is vital. While normal joint loading and exercise can be beneficial, excessive mechanical forces can lead to joint degeneration. Imaging, using [^18^F]NaF PET/MRI, can evaluate the tissue level joint response to loading, highlighting bone changes that precede cartilage damage in OA. Studies, in animals and humans, show that after exercise, [^18^F]NaF PET/MRI reveals localized, load-related bone responses, providing insights into early OA progression. However, more research is needed to understand the link between the bone's response to exercise and joint loading. Hybrid PET/MRI holds promise for guiding OA management by elucidating how bone, soft tissues of the joint, and loading forces interact.

## Authorship

All authors have made substantial contributions to all of the following: (1) the conception and design of review, (2) drafting the article or revising it critically for important intellectual content, (3) final approval of the version to be submitted.
